# O2.4. THE MISSING PIECE IN THE PUZZLE: COGNITIVE DECLINE IN SCHIZOPHRENIA AND BIPOLAR PATIENTS AFTER THE FIRST EPISODE

**DOI:** 10.1093/schbul/sby015.194

**Published:** 2018-04-01

**Authors:** Abraham Reichenberg, Jolanta Zanelli, Josephine Mollon, Sven Sandin, Paola Dazzan, Craig Morgan, Izabela Pilecka, Tiago Reis Marques, Gillian Doody, Kevin Morgan, Paul Fearon, Anthony David, Peter Jones, Robin Murray

**Affiliations:** 1 Icahn School of Medicine at Mount Sinai; 2 Institute of Psychiatry, Psychology and Neuroscience, King’s College London; 3 Psychiatric Imaging Group, MRC Clinical Sciences Centre, Institute of Clinical Science, Imperial College London; 4 University of Nottingham; 8 University of Westminster; 5 St. Patrick’s University Hospital and Trinity College Dublin; 6 University of Cambridge

## Abstract

**Background:**

Schizophrenia is associated with a severe cognitive impairment. While it is widely believed that cognitive deficits in schizophrenia remain stable after illness onset, few studies have comprehensively examined longer-term cognitive change from soon after the first episode through to late adulthood. We examined whether schizophrenia patients experience cognitive decline following the first episode, whether this decline is generalized or confined to individual neuropsychological functions, and whether decline is specific to schizophrenia.

**Methods:**

Participants were from a population-based, case-control study of patients with first-episode psychosis followed prospectively up to 10 years post first admission. A neuropsychological battery was administered at index presentation and at follow-up to patients with a diagnosis of schizophrenia and bipolar disorder or mania (n=83), as well as to healthy comparison subjects (n=103).

**Results:**

The schizophrenia group exhibited declines in IQ and individual neuropsychological functions tapping verbal knowledge, executive function, language and visual memory (group by time interaction p values<0.01). The age when progression of deficits where observed differed between functions. There was no decline in verbal memory and processing speed. These functions showed large deficits at the first episode, which remained static thereafter. Cognitive decline in IQ, verbal knowledge and language was not specific to schizophrenia and was also apparent in the bipolar/mania group (p values<0.05). Healthy individuals with low IQ, on the other hand, showed no evidence of decline, suggesting that a progressive course of cognitive impairment is specific to psychosis.

**Discussion:**

Schizophrenia and bipolar/mania patients experience cognitive decline after onset of psychosis. Cognitive remediation efforts should target individual functions during specific time periods.

